# Aluminum uptake and migration from the soil compartment into *Betula pendula* for two different environments: a polluted and environmentally protected area of Poland

**DOI:** 10.1007/s11356-015-5367-9

**Published:** 2015-09-15

**Authors:** Marcin Frankowski

**Affiliations:** Depatment of Water and Soil Analysis, Adam Mickiewicz University in Poznań, Umultowska 89b, 61-614 Poznań, Poland

**Keywords:** Aluminum, Fractionation, Soils, *Betula pendula*, Chemical plant, Wielkopolski National Park

## Abstract

This paper presents the impact of soil contamination on aluminum (Al) concentrations in plant parts of *Betula pendula* and a possible way of migration and transformation of Al in the soil-root-stem-twig-leaf system. A new procedure of Al fractionation based on extraction in water phase was applied to obtain and measure the most available forms of Al in soils and *B. pendula* samples. In addition, total Al content was determined in biological samples and pseudo total Al content in soil samples collected under plant saplings, using atomic absorption spectrometry with flame atomization. A number of relations concerning the occurrence of Al and Ca in soils and plant parts of *B. pendula* (tap roots, lateral roots, stem, twigs, and leaves) were observed. Based on the research findings, the mechanism of Al migration from soil to the leaves of *B. pendula* can be presented. It was found that aluminum uptake may be limited in roots by high calcium concentration. The application of a new procedure based on the simple sequential extraction of water-soluble fractions (the most available and exchangeable fractions of Al) can be used as an effective tool for the estimation of aluminum toxicity in soils and plants.

## Introduction

Al is one of the four most common elements occurring in soils. It is one of the most important components of soils, and to the same extent as silicon, it determines the crystalline frame of soil minerals. Al toxicity is the subject of many papers and reviews (e.g., Berthon 1996; Nayak 2002; Willhite et al. 2014), and research on Al impact on the environment is commonplace. The main symptom of Al toxic effects is the dramatic inhibition of root growth, which occurs within minutes after exposure to Al, even at micromolar concentrations (Tanaka et al. 1987; Illėš et al. 2006; Tolrà et al. 2011; Morita et al. 2011). Al toxicity is considered as one of the main factors limiting plant growth in acid soils (comprising about 40 % of the world’s arable lands) (Rout et al. 2001; Rezaee et al. 2013; Kovácik et al. 2012; Iqbal 2014). The speciation of Al in soils is a key factor in its potential not only to plant toxicity but also to living organisms as well. Al toxicity in soils and plants is not well understood, and the mechanism of Al migration is most commonly associated with the organic acid ions of citrate and malate (e.g., Flaten 2002; Fransson et al. 2004; Nunes-Nesi et al. 2014). Several papers describe the impact of Al on plants based on the use of Al salts, and most of them mainly focus on the root and its growth (Yang et al. 2013; Kidd and Proctor 2001; Klug et al. 2011; Yan et al. 2012, Doshi et al. 2008). It has been found that the occurrence of Al in soils is strongly connected with Al in leaves and its speciation (e.g., Frankowski et al. 2013, Karak et al. 2015). Moreover, research on the fractionation of Al shows that water extract fraction is the most important one due to the availability of Al species and their possible toxic impact. However, it should be noted that the total content or pseudo total content is important as well (e.g., Milačič et al. 2012; Frankowski et al. 2013). The most frequently used single-step extraction methods for Al and other elements, including heavy metals or metalloids, include the deionized water method (for the water-soluble fraction). Taking toxicity into account and Al bioavailability and migration from soils to living organisms, the determination of possible pathways of the migration and accumulation of particular Al species seems necessary. Especially, the mobile Al species (cold- and warm water-soluble fractions) and the total concentration of Al should be under focus. Mobile Al fractions are fundamental to learn more about possible pathways of Al transport in plants. In previous studies, the single-step extraction procedure was used to fractionate Al in agricultural soils (Takeda et al. 2006), forest soils (Zhu et al. 2004), flood plain soils (Drabek et al. 2005), sedimentary rocks, soils from mining areas (e.g., Matúš et al. 2006; Kubová et al. 2005), and soils from areas polluted with Al (Frankowski et al. 2010; Frankowski and Zioła-Frankowska 2010, Frankowski 2012).

The objectives of this research are to (1) develop the procedure for fractionation of Al in biological samples, based on water extract fractions, (2) determine the impact of soil contamination on Al concentration in the plant parts of *Betula pendula*, (3) determine the correlation of Al vs. Ca in soils and particular plant parts of *B. pendula*, and (4) determine the possible pathway of migration and transformation of Al in the soil-root-stem-twig-leaf system.

## Materials and methods

Soil and sapling (*B. pendula*) samples were collected for the analysis during September 2013. The samples of *B. pendula* (around 3-year-old trees) were divided into five plant parts: (1) twigs, (2) stems, (3) tap roots (without soil particles—removed manually), (4) lateral roots (root and root caps, without soil particles—removed manually), and (5) leaves.

**Fig. 1:**
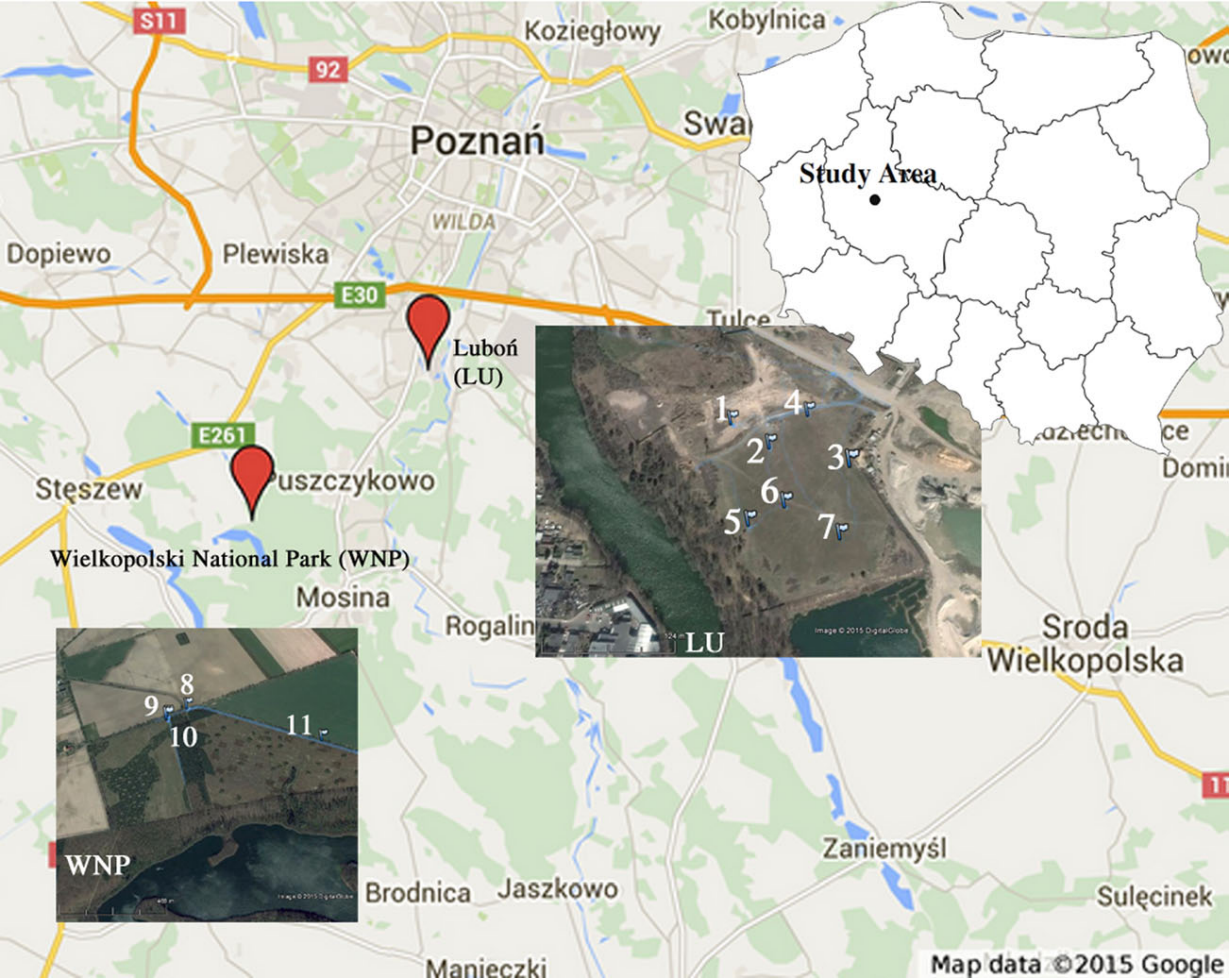
Sampling sites (Luboń Chemical Plant (LU) and Wielkopolski National Park (WNP))

To determine the spatial variability in Al content/concentration, the study area was divided into two “critical” areas with different Al concentrations in the soils—Luboń Chemical Plant (LU) and Wielkopolski National Park (WNP) (Fig. 1, Table 1).

**Table 1:** Sampling points

Sample no.	Site	Coordinates		Sample collected (separated fractions)
		N	E	
1	LU	52° 19′ 33.48″	16° 53′ 43.22″	
2	LU	52° 19′ 32.44″	16° 53′ 45.66″	
3	LU	52° 19′ 31.66‴	16° 53′ 50.47″	Twigs (F1, F2, TC)
4	LU	52° 19′ 33.88″	16° 53′ 47.84″	Stem (F1, F2, TC)
5	LU	52° 19′ 29.40″	16° 53′ 44.89″	Tap roots (F1, F2, TC)
6	LU	52° 19′ 30.08″	16° 53′ 46.82″	Lateral roots (F1, F2, TC)
7	LU	52° 19′ 28.90″	16° 53′ 49.96″	Leaves(F1, F2, TC)
8	WNP	52° 16′ 32.19″	16° 46′ 32.29″	Soils (F1, F2, PTC)
9	WNP	52° 16′ 30.56″	16° 46′ 27.02″	
10	WNP	52° 16′ 29.74″	16° 46′ 27.02″	
11	WNP	52° 16′ 25.40″	16° 47′ 11.50″	

LU is located in the southeastern part of the town of Luboń, 2.5 km south of the artificial recharge well field “Dębina” for the city of Poznań (Poland). The plant takes up the area of about 59 ha. To the north of the chemical plant, there are industrial grounds and afforested areas spreading across the meanders of the *Warta* River. To the south, there is an aggregate mine, and in a distance of about 0.7 km runs the border of WNP. In a distance of about 200 m to the southwest of the chemical plant, there is a post-crystallization leachate disposal site. The post-production disposal facility is a dammed-up underground tank with a built-up superstructure, which does not contain any additional safety devices to reduce the migration of pollutants to the water-bearing layer. It takes up the area of about 2 ha, and its removal began in the year 2005. The chemical plant produced aluminum fluoride for about 20 years. Post-crystallization leachate generated in the production process was stored at the post-crystallization leachate disposal site in the form of semi-fluid pulp. In the 1980s, such chemicals as superphosphate, hydrofluoric acid, aluminum fluoride, potassium fluoroborate, and vanadium catalyst were also produced there.

Soil samples for the analysis were collected in a depth profile of 0–20 cm. Samples were dried at room temperature. Hygroscopic substances dissolved in water were treated as an integral component of the samples. After drying, each soil sample was sieved through 2.0-, 1.0-, 0.5-, 0.25-, 0.1-, and 0.063-mm mesh size sieves, in accordance with the Polish Standards: PN-ISO 565:2000 and PN-ISO 3310-1:2000, using a LAB-11-200/UP sieve shaker (EKO-LAB, Brzesko, Poland). The grain size fraction between 0.1 and 0.25 mm was predominant and was used to prepare soil extracts. Leaf samples were ground, and the other parts of the trees were divided into 0.5-cm pieces. Subsequently, these samples were stored in polypropylene bags until extraction and mineralization. All the 0.5-cm pieces of particular plant parts were used for extraction.

Fractionation of Al and Ca in the water fraction of soils and biological samples:

- Fraction F1: mobile Al obtained by water extraction at room temperature (25 °C)—the most available and mobile form of Al, environmentally available (Frankowski & Zioła-Frankowska 2010; Frankowski et al. 2013)
- Fraction F2: bound residue of F1—obtained by extraction at 80 °C. An available and partially exchangeable form of Al (weakly bound to structural parts of biological samples and partially bound on the surface of grains in soil samples)
- TC: total content of structural and mobile forms of Al and Ca in biological samples. Total content in the case of Ca in soils and pseudo total content (PTC) in the case of Al in soils

The F1 extracts were prepared in a 1:10 (*v*/*v*) proportion with deionized water. They were homogenized during 1 h in a magnetic mixer (the predominant 0.1—0.25-mm grain size fraction was used for the soil samples). Subsequently, the F1 extracts were placed in falcon tubes and a new portion (10 ml) of deionized water was added. During that step, the extraction temperature was increased to 80 °C (hence named fraction F2). The pH was determined for all the water extracts, using an Orion 5-star Plus (Thermo, USA) meter with a Single Pore pH electrode (Hamilton, USA). In the next step, the soil samples were mineralized using a modified EPA 3051 method (Frankowski et al. 2013) to separate the pseudo total concentration (PTC) of Al and the total concentration (TC) of Ca. The same method was used for the mineralization of biological samples. Measurements of Al and Ca were performed in three replications, and the relative standard deviation did not exceed 7 %. The elements were determined using a Shimadzu AA7000 spectrometer (Shimadzu, Japan), with an air-acetylene flame atomization for Ca and with a nitrous oxide-acetylene flame atomization for Al.

To check the accuracy of the flame atomic absorption spectrometry analytical technique, a standard procedure making use of certified reference materials was adopted:

- The SRM 2709—for soils (soil samples prepared in accordance with EPA method for FAAS)
- The SRM 1515—for leaves (National Institute of Standards and Technology, USA)

The SRM 2709 and SRM 1515 reference materials were analyzed in six replications. Average recoveries for all the measured elements in both SRM materials were within range of the certified concentrations.

## Results and discussion

Research on Al fractionation using single-step extraction and simple sequential extraction of soils and particular plant parts of *B. pendula* indicated the requirement to investigate the issue of Al availability and bioavailability. This subject has to date been included in the studies by Drabek et al. (2005), Álvarez et al. (2002), Walna et al. (2005), and Zołotajkin et al. (2011). Soil samples most frequently originate from highly acidified areas. Moreover, it should be emphasized that knowledge about Al availability and bioavailability, from soils through roots and finally to leaves, is important to determine possible toxic effects for plants and possible pathways of Al transport in the plant system.

### Total Al in *B. pendula*—detailed characterization for different plant organs

**Fig. 2:**
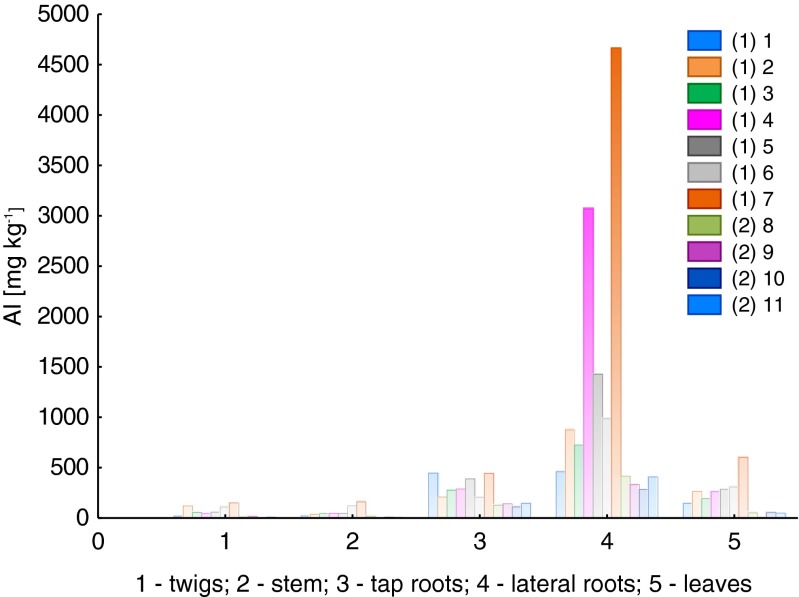
Total content of aluminum in particular parts of *Betula pendula* (*1–11*, samples *1* and *2* mean that they have been collected at the LU and WNP sites, respectively)

Figure 2 presents the results of total Al content for particular plant parts of *B. pendula*, for two different types of environments: Luboń Chemical Plant (LU) and Wielkopolski National Park (WNP).

The highest content of Al was identified in lateral roots, especially in samples taken from the contaminated area (LU). Samples 4 and 7 had Al contents of 3.1 and 4.7 mg kg^−1^, respectively. The other samples ranged from 463.1 to 1429 mg kg^−1^ in the LU case and from 289.3 to 419.3 mg kg^−1^ in the WNP case. Relatively high Al concentrations were found as well in tap root samples taken from the LU area, ranging from 212.2 to 450.3 mg kg^−1^, and from the WNP area, ranging from 113.8 to 145.1 mg kg^−1^. Based on results of Al measurements in tap roots, we could observe a clear difference between samples, depending on the sampling site (LU or WNP). A similar phenomenon was found for leaves as well, where Al seems to accumulate. In the samples from the contaminated area (LU), Al content in leaves ranged from 147.3 to 605.7 mg kg^−1^. The highest Al content in leaves was determined for sample 7 (also the highest Al content in lateral root). As for the samples taken from the WNP area, the content of Al ranged from 12.41 to 55.75 mg kg^−1^. Similar relations regarding Al content were found for the samples of other plant organs: twigs and stems. However, for these samples, Al content was much lower, respectively, in the range 23.5–153.8 mg kg^−1^ for twig samples taken in the LU area and 9.6–17.1 mg kg^−1^ for the samples taken from the WPN area. In the case of stem samples, Al content ranged from 24.6 to 165.3 mg kg^−1^ (LU site) and from 9.6 to 22.5 mg kg^−1^ (WNP site). It was observed that Al content in stems was lower than in lateral roots (LU site), while for samples collected in the WNP site, the dependence of Al content in stems and lateral roots was not clear; 2 out of 4 samples had higher Al contents in stems than in lateral roots.

### PTC of Al in soils vs. Al in *B. pendula*

Pseudo total content (PTC) of Al represents the fraction occluded on the grains of soils and not bound to the soil crystalline structures. Table 2 presents the concentrations of Al for the 0.1–0.25-mm grain size fraction and the percentage of F1 and F2 fractions in PTC.

**Table 2:** Pseudo total content (PTC) of aluminum (mg kg^−1^) and the percentage of F1 and F2 fractions in PTC of aluminum in soil samples

sample no.	PTC	F1	F2	F1 (%) of PTC	F2 (%) of PTC
1 (1)	2522	72.03	106.3	2.856	4.214
2 (1)	2472	278.7	351.1	11.27	14.20
3 (1)	4431	473.2	569.7	10.67	12.85
4 (1)	3498	225.2	309.6	6.437	8.851
5 (1)	6148	426.4	501.9	6.935	8.163
6 (1)	4640	450.9	409.6	9.717	8.828
7 (1)	1501	248.1	144.7	16.52	9.640
8 (2)	1651	29.88	58.72	1.810	3.556
9 (2)	1319	7.716	39.41	0.584	2.987
10 (2)	1985	2.574	24.49	0.130	1.233
11 (2)	1395	4.714	18.89	0.337	1.354

Samples 1 and 2 mean that they have been collected at the LU and WNP sites, respectively

**Fig. 3:**
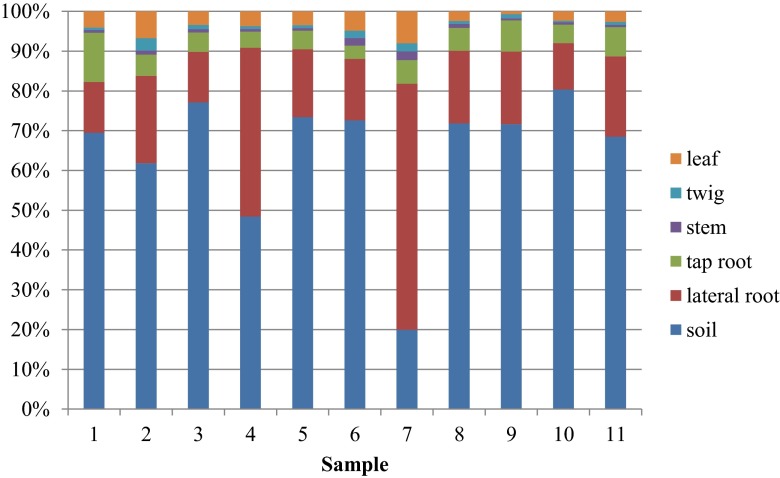
Percentage of aluminum in soils and particular plant parts of *Betula pendula*

PTC for the samples collected in the LU area was variable and averaged to a value of 3601 mg kg^−1^. The highest concentration was found for sample 5. For the WNP area, Al concentrations were similar, with an average value of 1588 mg kg^−1^. Still, the contribution of fractions F1 and F2 to PTC varied for the samples taken in the LU area. They amounted to 2.9–16.5 % for the F1 and 4.2–14.2 % for the F2 fraction. Samples of soil taken in the WNP site varied as well and amounted to a range of 0.13–1.81 % for the F1 fraction and 1.23–3.56 % for the F2 fraction. The uptake of Al, when based on the PTC of Al in soils and particular plant organs of *B. pendula*, is presented in Fig. 3.

Based on the percentage share of Al PTC in soil and TC in *B. pendula*, it was observed that, except for samples 4 and 7, the proportions were similar in the LU and WNP areas. It means that the content of Al in particular plant parts did not depend on soil contamination. The uptake of Al, which was strongly connected with the concentration of Al in leaves, was evenly distributed in *B. pendula* plant parts, and it was limited by the root system.

### F1 in *B. pendula*—detailed plant organ characterization

**Fig. 4:**
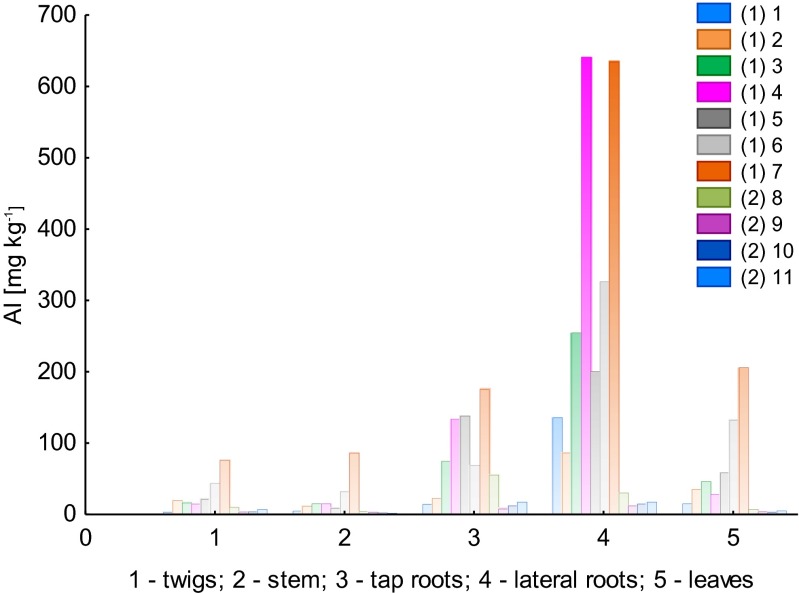
Concentration of aluminum in the F1 fraction of the particular plant parts of *Betula pendula* (*1–11*, samples *1* and *2* mean that they have been collected at the LU and WNP sites, respectively)

Figure 4 presents Al concentration in the F1 fraction, for particular plant parts of *B. pendula* and in two different environment types: LU and WNP.

The Al concentration in the F1 fraction demonstrated a similar variability as total content of Al. Fraction F1 is the most mobile fraction of Al, and considering the samples taken at the LU site versus those taken at the WNP site, it can be noted that the availability of Al for the two areas was different. This refers particularly to the results obtained for leaf samples, in which the Al concentration was much lower for the WNP site. It can be emphasized as well that Al concentrations were highest in the lateral and tap roots when compared with the other plant parts of *B. pendula*. This was confirmed by the results for the TC fraction. For lateral root samples, Al concentrations (in the F1 fraction) of 86.2–640.6 and 12.0–30.3 mg kg^−1^ were determined, whereas for tap roots, the ranges were as follows: 14.0–175.1 and 7.9–55.5 mg kg^−1^ for the LU and WNP sites, respectively. Al concentrations in leaves ranged from 15.0 to 205.8 mg kg^−1^ for samples collected at the LU site and from 2.8 to 7.1 mg kg^−1^ for samples collected in the WNP area. Regarding twigs and stems, the concentration of Al was lower for stem samples. The concentration ranges were as follows: twigs 3.0–76.0 (LU site) and 3.4–10.1 mg kg^−1^ (WNP site) and stems 4.9–86.1 mg kg^−1^ (LU site) and 2.1–4.5 mg kg^−1^ (WNP site).

### F2 in *B. pendula*—detailed plant characterization

**Fig. 5:**
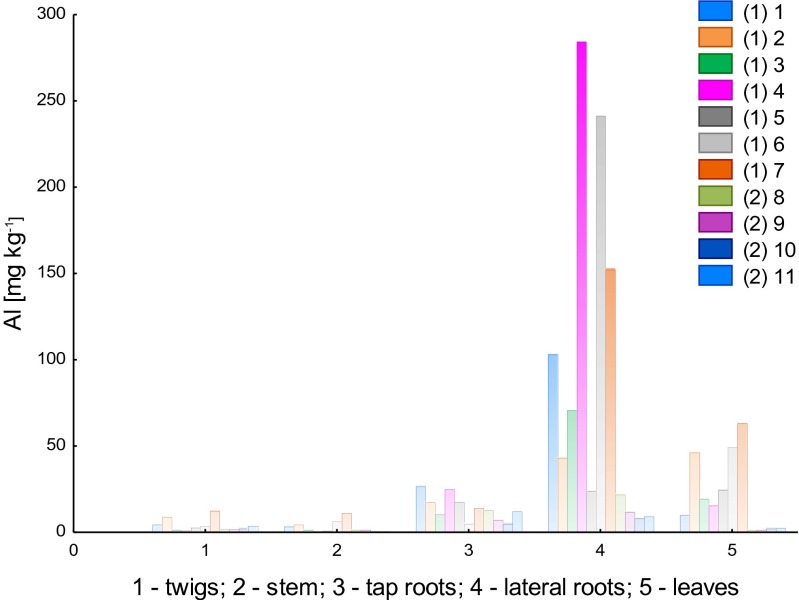
Concentration of aluminum in the F2 fraction of particular plant parts of *Betula pendula* (*1–11*, samples *1* and *2* mean that they have been collected at the LU and WNP sites, respectively)

Figure 5 presents Al concentration in the F2 fraction, for particular plant parts of *B. pendula* and in two different environment types: LU and WNP.

**Fig. 6:**
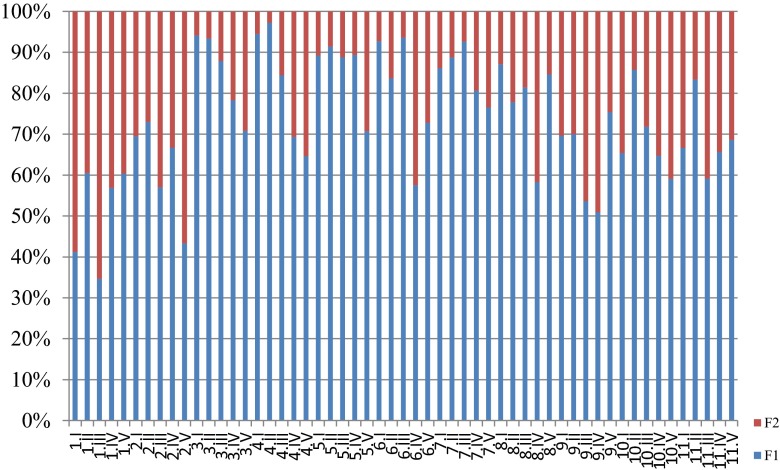
The percentage of F1 and F2 fractions (symbol *1.I* means sample.plant part: *I* twigs, *II* stem, *III* tap roots, *IV* lateral roots, and *V* leaves)

Low Al concentration in F2 samples of twigs and stems indicated the transport of ions in these plant parts and the accumulation of Al in leaves. For the F2 fraction, the Al concentration in leaves was as follows: 1.3–2.4 mg kg^−1^ for WNP samples and 9.9–63.1 mg kg^−1^ for samples taken at the LU site. Such a variation of Al concentrations in the two environment types (LU vs. WNP) suggests the accumulation of Al with time and binding of Al to soluble complexes which are not extracted by water during F1 extraction. A similar relation was found for lateral root samples, for which the F2 fraction Al concentrations were as follows: 7.9–21.7 mg kg^−1^ for the samples taken at the WNP site and 23.8–283.1 mg kg^−1^ for the LU site samples. It was observed that the binding of Al by the specific plant parts was much higher for the samples collected from the LU site than for the samples taken at the WNP site. This suggests the continuous accumulation of Al during the vegetation season. To determine the degree of Al binding, the % value of fraction F1 versus F2 is presented in Fig. 6.

The % of fraction 1 versus fraction 2 varied, especially for samples 1 and 2 taken at the LU site, in which a higher extraction degree was observed for the F2 fraction than for the other samples taken from that site. Besides, the extraction degree for samples 1 and 2 indicated complex mechanisms of Al binding to structures of particular plant parts. For samples 3 to 7, the F1 versus F2 variability was similar and the highest % of the F1/F2 ratio was found in the samples of lateral roots and leaves. In the case of samples taken at the WNP site, the variability in F1/F2 percentage was similar and the tendency for particular plant parts was comparable.

### Bioavailability of Al (TC vs. F1)

**Fig. 7:**
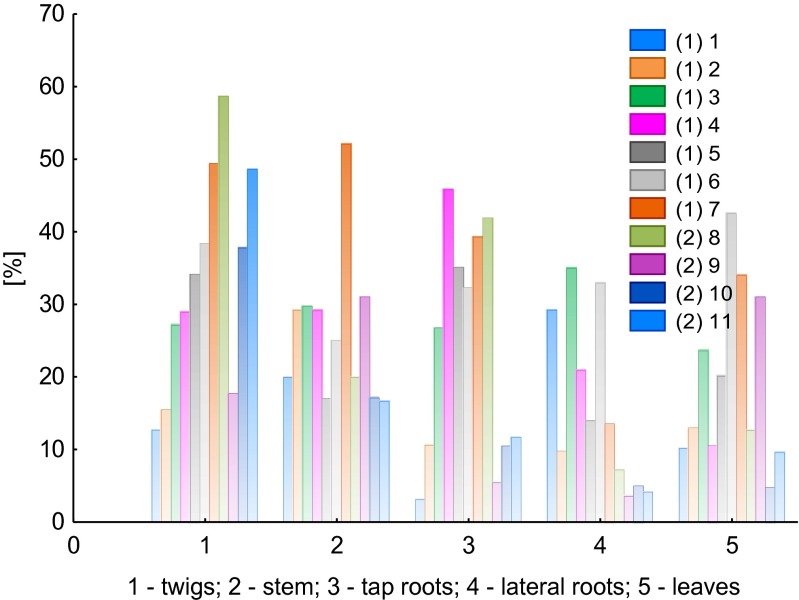
The percentage of fraction F1 with respect to the total content of aluminum in particular plant parts of *Betula pendula* (*1–11*, samples *1* and *2* mean that they have been collected at the LU and WNP sites, respectively)

The percentage F1 fraction versus TC reflects the availability of Al which has been transported from the root system to the leaves of *B. pendula*. The variable F1/TC also indicates the concentration of mobile Al which is subject to transformations (especially concerning its chemical forms) and contributes to the toxicity of this element. Figure 7 presents the % share of F1 in total content of Al for particular plant parts of *B. pendula.*

Based on the F1/TC values of Al, it is difficult to pinpoint a relationship between the results obtained for the LU and WNP sites. This particularly pertains to the values obtained for the samples of twigs and stems. The degree of extraction for these plant parts was variable: 12.7–49.4 and 17.8–58.8 %, respectively, for the LU and WNP site twig samples and 17.1–52.1 and 16.7–31.1 % for the LU and WNP site stem samples. In the other plant parts, i.e., the lateral roots and tap roots, a lower degree of the extraction of F1 in relation to TC was observed, especially for samples 9–11 (WNP site). In the case of leaf samples, it was observed that variability was low in the extraction efficiency, indicating the presence of weakly bound Al, e.g., organic Al complexes.

### The impact of pH

**Fig. 8:**
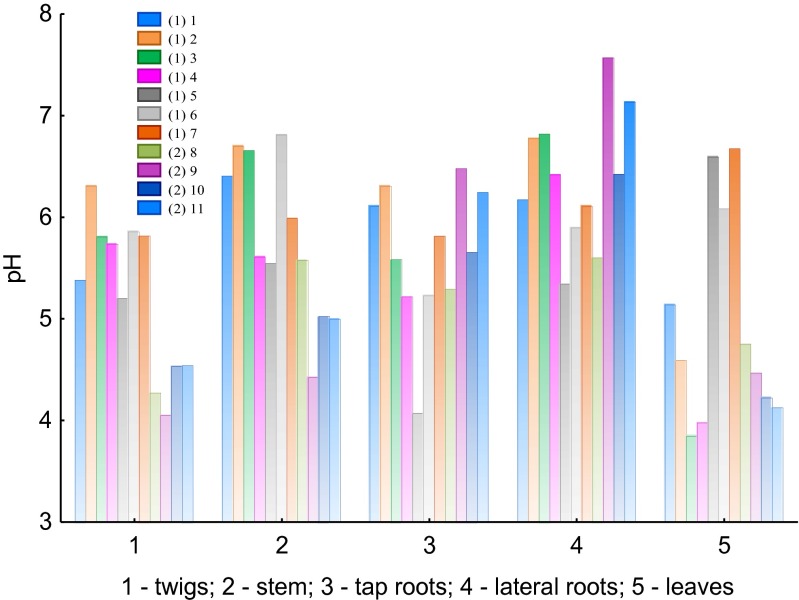
pH values for particular plant parts of *Betula pendula* samples in the F1 fraction (*1–11*, samples *1* and *2* mean that they have been collected at the LU and WNP sites, respectively)

The concentration of [H^+^] ions in the F1 fraction for all samples (different plant parts) was similar for the WNP and LU sites (Fig. 8). However, pH variability in samples of leaf water extracts, taking values from 3.9 to 6.7 for the LU site and from 4.1 to 4.8 for the WNP site, should be further discussed.

Low pH values in leaf samples can be explained by the occurrence of organic acids in leaves, which—depending on the concentration of ligands (e.g., oxalate, citrate, malonate, acetate, formate)—can lower Al toxicity by the formation of relatively stable Al complexes with a considerable advantage of the ligand/Al^3+^. Expressed toxic impacts at low contributions of the ligand/Al^3+^ are a consequence of low ligand occurrence (e.g., Frankowski et al. 2013).

### TC of Ca vs. Al in soils and *B. pendula*

The total content of Ca was higher in samples of *B. pendula* than in soils. The variability of Ca concentrations in particular plant parts was similar to that of Al concentrations (for both the F1 and F2 fractions and PTC). This relationship indicates a strong connection between the occurrence of Ca and Al in soils as well as similar mechanisms of uptake by the plant root system and transport to leaves. Table 3 presents the TC of Ca determined for particular plant parts and the concentration of Ca in soils.

**Table 3:** Total content of Ca (mg kg^−1^) in particular plant parts of *Betula pendula* and in soil samples

Sample no.	Twig	Stem	Tap root	Lateral root	Leaf	Soil
1 (1)	4844	6232	9767	9094	9146	2075
2 (1)	7942	4367	5242	6611	9812	2712
3 (1)	6677	3887	7446	6562	7343	3324
4 (1)	6168	4733	6455	7794	8275	2540
5 (1)	5210	4300	7020	8049	7683	5211
6 (1)	5963	5531	6076	5962	9281	3636
7 (1)	4063	3916	5714	8691	11,930	628.4
8 (2)	4942	2845	5756	5518	7413	556.1
9 (2)	2952	2245	2818	3467	4896	294.9
10 (2)	3387	2169	3212	3981	6278	957.7
11 (2)	3067	2590	5115	5811	5782	334.7

Samples 1 and 2 mean that they have been collected at the LU and WNP sites, respectively

**Fig. 9:**
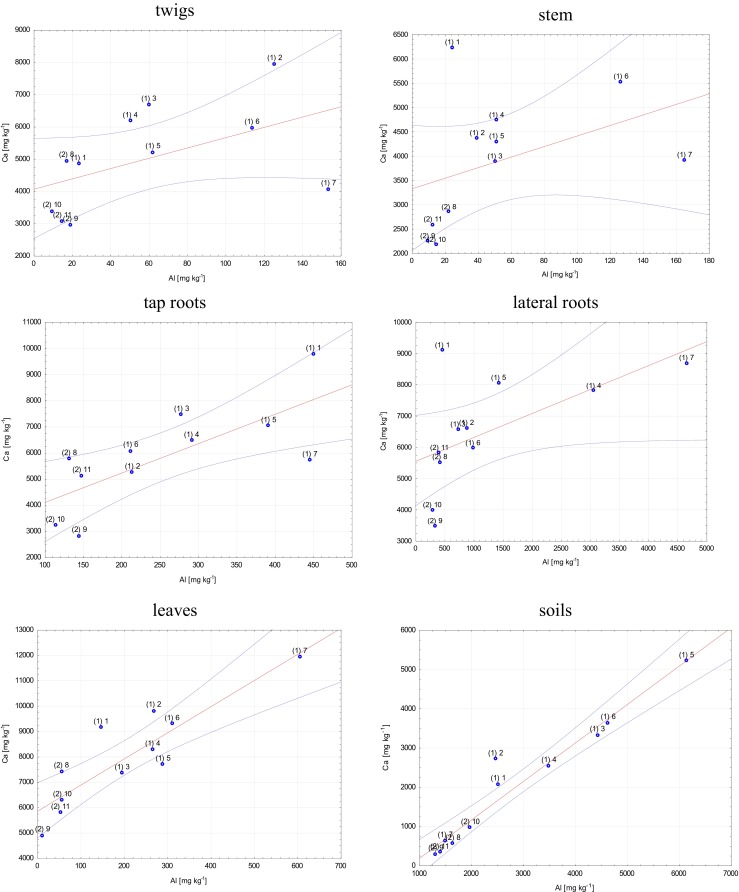
Graphs representing the correlations of Al and Ca concentrations for twigs, stem, tap roots, lateral roots, leaves, and soil samples (significance level *p* = 0.95)

To determine and open the discussion on the dependences of the occurrence of Ca and Al in soils and in particular plant parts, correlations are presented for each group of samples (Fig. 9).

The results obtained for soil samples showed that concentrations of Al were highly dependent on the concentration of Ca (*r* = 0.969). This allows us to state that the availability of Ca and Al cations was regulated in the soil. Ca is taken up from soils through the root system, first, through the lateral roots and, subsequently, by the tap roots. With regard to the relationship between Al and Ca in lateral roots, it was found that Al^3+^ cations were “blocked,” and as a result, Ca^2+^ cations were introduced. First of all, this can be explained by the much lower concentration of Ca in the lateral root system than in tap roots, combined with the retention of Ca in the root system. In this case, lateral roots prevented the introduction of Al to tap roots and with respect to transport, further to the stems and twigs and finally to the leaves. The process was limited by the uptake of higher amounts of Ca^2+^ by the system. This was confirmed by the results of the Ca/Al correlation for tap roots. We obtained much higher values of coefficient *r* = 0.736 as compared to later roots. On that basis, it can be assumed that Al does not migrate from soils to roots as Al^3+^ but in other speciations, i.e., Al complexes which are both of inorganic and organic nature and with a different charge (e.g., +1, −1, and +2, −2). Also emphasized is that the correlation study results for tap roots showed that *B. pendula* plants are able to regulate the concentration of Ca versus that of Al; this was evident for both the samples taken from the LU site and those from the WNP site. The relationship between Al and Ca in stem and twig samples was disturbed, which might be connected with the transport of the cations toward the leaves. The results of Ca and Al concentrations for leaves and the correlation between these elements (*r* = 0.882) showed that Al can easily migrate from tap roots to leaves. A specific amount of Al was built in as a structural element, and a considerable amount was transported to the leaves (see Fig. 5). Taking the pH of the water extracts of leaf samples into account (Fig. 8), it can be noted that Al species can be transformed, especially to the Al^3+^ species. The toxic impact of this Al form is probably lowered by the availability of organic ligands (e.g., malate, oxalate, or citrate ions). As it is commonly known, organic complexes of Al are much less toxic or not toxic at all for plants or living organisms. Similar relationships between Al and Ca concentrations were observed for both the F1 and F2 fractions.

Kidd and Proctor (2001) conducted research on the impact of Al on growth and mineral composition of *B. pendula* Roth and concluded that low Al concentrations (2 and 5 mg l^−1^) enhanced growth, whereas higher Al concentrations (10–15 mg l^−1^) reduced growth in less Al-tolerant plant races.

### Statistical analysis

The statistical analysis of Al concentrations in the F1 and F2 fractions as well as the TC of Al in soils and particular plant parts of *B. pendula*, based on the Kolmogorov-Smirnov test, did not give ground to reject the hypothesis on the equality of means in the studied groups of samples. Similarly, the Shapiro-Wilk test indicated that 11 out of 18 types of soil samples and plant parts (F1, F2, and TC fractions) were characterized by a normal distribution. The samples of tap root F1 and soil F1 fractions as well as tap root F2 and the TC fractions of twig, tap root, leaf, and soil samples (in total, 7 out of 18 types) were not normally distributed (level of significance *p* < 0.05). To compare the concentration of Al in the study sites (LU vs. WNP), the *U* Mann-Whitney test was executed. For Al in soils and *B. pendula*, the obtained values of *p* were lower than *α* = 0.05, except for twig F1 fraction and twig, stem, and tap root F2 fractions. These results led to the conclusion that the Al concentrations in the F1, F2, and the TC fractions of Al were statistically significantly different for both the investigated sample sites (LU and WNP).

## Conclusions

The application of a new procedure, based on the simple sequential extraction of water-soluble fractions (F1 and F2), can be used as an effective tool for the estimation of soil Al toxicity in plants. Moreover, it can be emphasized that data on total (plants) and pseudo total (soil) content of Al are important indeed and should always be taken into account when performing Al (toxicity) research. Additionally, it can be concluded that the proposed procedure is useful in evaluation of the distribution of Al in soils and plants. It was elicited that in the samples originating from the LU site, binding of Al by particular plant parts is much higher than in the samples taken from the WNP sampling site. This suggests a persistent accumulation of Al during the growing season. The concentration of Ca in plants and soils was used to understand the mechanism of Al migration from soil to leaves through the plant system. It was observed that the variability of Al in particular plant parts and the concentration of Al do not depend on soil contamination. However, based on a statistical analysis, the differences between samples collected from the LU and WNP were indicated. The uptake of Al was evenly distributed in *B. pendula* plant parts and was limited by the root system as well as strongly connected with the concentration of Al in leaves.

## Abbreviations

LU: Luboń Chemical Plant
WNP: Wielkopolski National Park
PTC: Pseudo total content
TC: Total content
F1: Water fraction 1
F2: Water fraction 2
FAAS: Flame atomic absorption spectrometry
